# The effects of cervical spine posture on susceptibility to walking balance perturbations

**DOI:** 10.1371/journal.pone.0354125

**Published:** 2026-08-05

**Authors:** Emily K. Eichenlaub, Aaron Gelinne, Deb Bhowmick, Jason R. Franz

**Affiliations:** 1 Joint Department of Biomedical Engineering, University of North Carolina Chapel Hill and North Carolina State University, Chapel Hill, North Carolina, United States of America; 2 Department of Neurosurgery, University of North Carolina Chapel Hill and North Carolina State University, Chapel Hill, North Carolina, United States of America; 3 Department of Neurosurgery, Duke University, Durham, North Carolina, United States of America; Johns Hopkins: Johns Hopkins University, UNITED STATES OF AMERICA

## Abstract

**Background:**

Degenerative cervical myelopathy (DCM) is the most common cervical spine disorder encountered in the aging population that commonly presents with increased cervical kyphosis and impaired gait. Thus, the compounding effects of exaggerated head tilt, as a result of cervical deformity, may increase the risk of falling in individuals with DCM.

**Research question:**

What are the effects of cervical bracing and posture (i.e., kyphosis and lordosis) on outcomes relevant to the control of walking balance?

**Methods:**

This was an experimental study conducted in 15 healthy young adults. We used two discrete mechanical balance challenges designed to elicit walking-related instability. Specifically, participants walked with a series of cervical spine braces while responding to: (i) treadmill-induced slip perturbations to induce rapid reactive responses to unanticipated perturbations and (ii) a reactive lateral stepping as a goal-directed balance challenge. We used two-way repeated measures ANOVAs to determine the effect of bracing condition and balance challenges on (i) anterior-posterior and mediolateral margins of stability (MoS_AP_ and MoS_ML_) and (ii) reaction time and foot placement error.

**Results:**

Our experimental manipulations successfully emulated cervical spine postures common to patient populations. Compared to unperturbed walking, treadmill belt decelerations elicited significantly smaller MoS_AP_ and larger MoS_ML_ for all conditions, but elicited negative MoS_AP_ indicative of instability only for braced conditions. Bracing did not increase foot placement errors during lateral reactive stepping compared to unbraced walking. However, only for kyphotic bracing did performance decrease with target distance.

**Significance:**

Individuals prescribed cervical immobilization or presenting with cervical deformities may be less capable of responding to balance challenges that could precipitate a fall in the community. We conclude that treating cervical spine deformities with cervical immobilization may benefit from educational materials and monitoring techniques to mitigate falls risks.

## Introduction

Degenerative cervical myelopathy (DCM) is the most common cervical spine disorder encountered in the aging population [1]. Falls are disproportionately common among this group, thus accounting for a significant cost and resource burden to the healthcare system [2]. Individuals with DCM who experience falls are also at a greater risk of spinal cord injury and lifelong debilitation [3,4]. Due to progressive compression of the spinal cord, individuals with DCM present with neurological deterioration which commonly manifests as impaired gait [5,6]. Specifically, these individuals have reduced stride length, stride velocity, and prolonged stance phase duration compared to healthy controls [7,8]. Moreover, increased cervical kyphosis is a typical abnormal cervical alignment parameter observed in the setting of DCM [9]. Cervical kyphosis and spinal cord compression commonly co-exist and are rarely present independently [1,9]. Thus, the compounding effects of exaggerated head tilt as a result of cervical deformity may increase the risk of falling in individuals with DCM.

Cervical deformity can be characterized by the degree of kyphosis (forward bending) or lordosis (backward bending) of the cervical spine [10]. The degree of kyphosis and lordosis has been correlated to overall health scores and myelopathy severity [10–12]. Additionally, patient-reported outcomes suggest that changes in cervical spine posture from values considered normal are associated with increased functional impairment and fall risk [4,12].

While perturbation-based balance training has been applied [13], and perturbation-based assessments have characterized balance impairments in individuals with DCM [14,15], the effects of cervical posture on balance has yet to be objectively quantified, even in otherwise healthy individuals. Those with DCM often have comorbidities which can confound the relation between cervical posture and walking balance integrity. Thus, a critical first step that has yet to be taken is to study the effects of cervical posture on susceptibility to walking balance perturbations in healthy controls. Additionally, the influence of cervical immobilization itself on deformities is a longitudinal issue that can be challenging to observe and quantify in a well-controlled study. This becomes especially relevant in clinical practice where the only definitive treatment for DCM is surgery to decompress the spinal canal. Some studies have advocated for the restoration of cervical lordosis [16]. However, successful correction of cervical posture via surgical intervention is less definitive and a matter of controversy [17,18]. To this end, more objective data on cervical posture as it relates to walking balance is needed in order to guide clinical decision making.

Therefore, the purpose of this study in healthy young adults was to investigate the effects of cervical bracing and posture (i.e., kyphosis and lordosis) on outcomes relevant to the control of walking balance. We used two discrete mechanical balance challenges designed to elicit instability during walking. Specifically, we used treadmill-induced slip perturbations to induce rapid reactive responses to unanticipated perturbations and reactive lateral stepping as a goal-oriented balance task. We hypothesized that induced cervical deformities (i.e., kyphosis and lordosis) would elicit larger susceptibility to treadmill-induced slip perturbations and reduced lateral reactive stepping performance compared to walking with full cervical mobility and neutral immobilization, respectively.

## Methods

### 2.1. Participants, experimental protocol, perturbations, and measurements

Fifteen healthy young adults were recruited to participate in this single-visit study (5 men/10 women; mean ± standard deviation; age: 24.3 ± 4.5 years; height: 1.72 ± 0.11 m; mass: 69.02 ± 10.83 kg). All participants confirmed absence of neurological disorders and lower extremity injuries or fractures within the last six months and could walk without an assistive device. Prior to data collection, participants provided written informed consent and the study was approved by the University of North Carolina Biomedical Sciences Institutional Review Board (Study **#**21-3091). Recruitment began April 15, 2022 and concluded June 18, 2022.

Four spinal immobilization conditions were investigated in this study: unbraced, neutral, kyphosis, and lordosis. Cervical kyphosis is an abnormal downward curvature of the neck, which some studies have measured to be an average of 45° in patients [19]. Cervical lordosis is an abnormal upward curvature, or extension, typically between 20–35° [20]. An adjustable universal cervical collar (Aspen Medical Products, Irvine, California, USA) was used for cervical immobilization and to artificially induce cervical kyphosis and lordosis. This collar has six adjustable height settings to change the neck angle, with 1 being the lowest and 6 being the highest. Therefore, the collar was set to 3 for the immobilized (braced) condition, 6 for the lordotic condition, and 1 for the kyphotic condition. A custom pad was inserted into back of the collar for the kyphotic position to ensure participants could not change their neck angle.

We first measured each participant's preferred overground walking speed (i.e., 1.31 ± 0.13 m/s) from the average of 4 times taken to walk 30 meters. We then placed 48 retroreflective markers on participants’ pelvis, trunk, arms, legs and head. Specifically, pelvis and lower limb markers were placed on the sacrum, bilateral anterior superior iliac spines, posterior superior iliac spines, lateral femoral epicondyles, lateral malleoli, lateral calcanei, and lateral first and fifth metatarsal heads; torso markers were placed on the sternum, clavicle notch, vertebra prominens, and tenth thoracic vertebra; upper extremity markers were placed on the bilateral acromia, lateral humeri, lateral humerus epicondyles, and radial and ulnar styloid processes; head markers were placed over a tight-fitting head cap on the crown, 50 mm superior to the external acoustic meatuses, and 50 mm superior to the lacrimal. Rigid tracking marker clusters were also affixed to participants’ thighs and shanks. In trials using the cervical collar, virtual markers to represent the C7 and clavicle were added in post-processing based on their positions in unbraced conditions.

**Fig 1:**
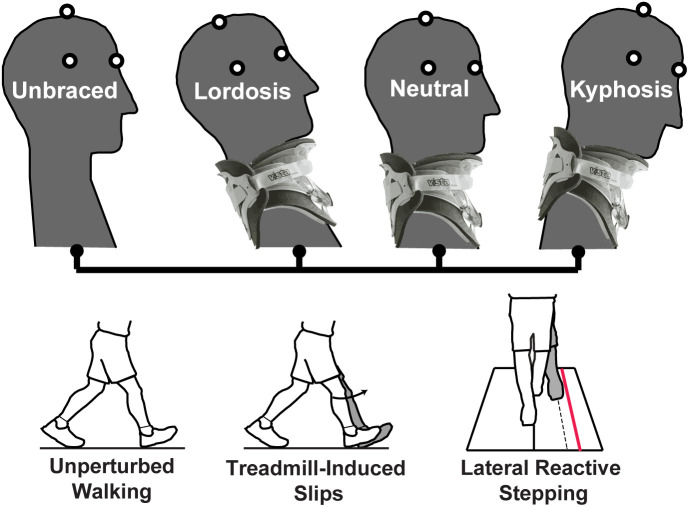
Experimental protocol for each perturbation paradigm and bracing condition. Participants were either in an unbraced or braced condition. Braced conditions included cervical immobilization or artificially induced kyphosis or lordotic neck postures. Within each bracing condition, participants completed 2 minutes of unperturbed walking, a series of treadmill-induced decelerations, and a lateral reactive stepping task in a randomized order.

Following marker placement, participants walked on a motor-propelled, split-belt treadmill (Bertec Instrumented Treadmill, ITC-11-20L-5; Bertec Corp., Columbus, Ohio, USA) for two minutes at their preferred overground walking speed for each of the four conditions. Participants then completed a block-randomized series of walking trials with the addition of two contexts of perturbations (Fig 1). We employed one perturbation paradigm to test the effects of cervical spine condition on walking instability. In this trial, participants responded to 200 ms, 6 m/s^2^ treadmill belt decelerations delivered at the instant of random heel strikes using a custom Matlab script (MathWorks, Natick, MA, USA). Following the 200 ms perturbation, the treadmill belt immediately returned to the participant’s preferred overground walking speed over 200 ms at the same 6 m/s^2^. Participants were informed that the treadmill would briefly slow to simulate a slip and were instructed to respond and return to normal walking as best as they can. A total of four perturbations were delivered to equal two decelerations on each leg. In agreement with previous literature, we define a deceleration, or slip, as a decrease in the posterior velocity of the foot being prescribed by the treadmill belt surface [21,22]. We also used one task designed to isolate a critical feature of walking balance control – lateral reactive stepping. In this task, participants performed a reactive stepping task by responding to lateral targets projected onto the treadmill surface using a custom-built computer-controlled projection system. Bilateral near and distant targets were displayed twice on either side of the treadmill in a randomized order. The target was triggered at the preceding ipsilateral heel-strike. Participants were instructed to step out to the target using the foot closest to the target on the following ipsilateral heel strike. All trials were included in the analysis.

### 2.2. Outcome measures

**Fig 2:**
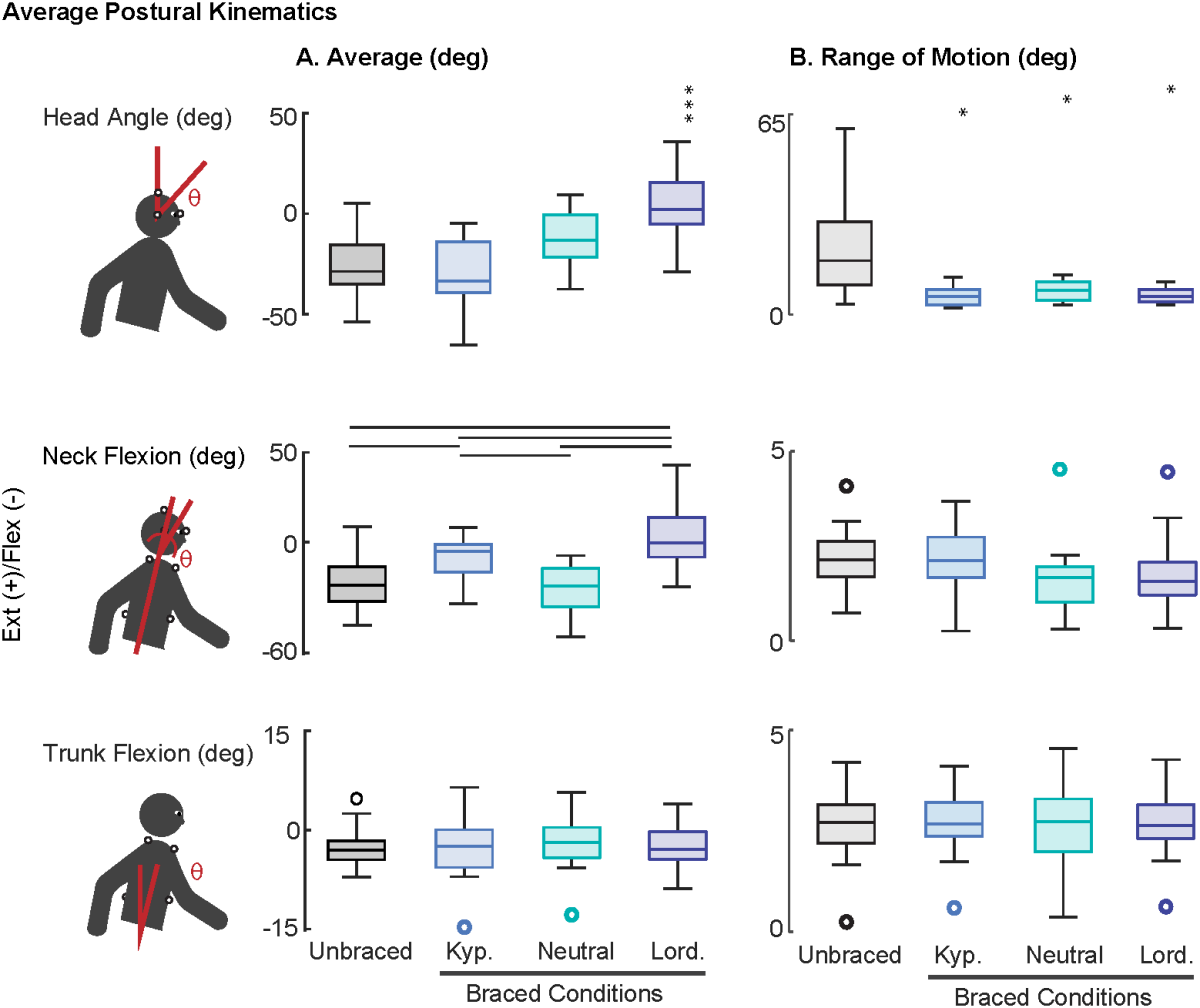
Average postural kinematics (i.e., head, neck, and trunk angles) and range of motion during normal, unperturbed walking for all four bracing conditions. Asterisks (*) denote significant differences between bracing conditions and the unbraced condition. Black bars denote significant differences between bracing conditions.

To validate our protocol was successful in artificially inducing lordosis and kyphosis, we calculated the average of and range of motion (ROM) the head, neck, and trunk flexion angles over the average unperturbed gait cycle in Visual3D (C-Motion, Rockville, MD, USA). The head segment was defined by the crown marker (most superior point on the head) and the markers placed 50 mm superior to the external acoustic meatuses (Fig 2), or the most superior point on the head. Global head angle was calculated as the angle between the head average head segment and the virtual laboratory’s vertical axis. Neck flexion was calculated as the angle between the head and trunk segments. The trunk segment was defined according to ISB recommendations based on the C7, clavicular notch, T10, and sternum marker positions [23]. Global trunk flexion was calculated as the angle between the trunk and the virtual laboratory’s vertical axis. We also calculated the average step width during unperturbed trials for all four bracing conditions.

We chose anterior-posterior Margin of Stability (MoS_AP_) to capture instability elicited by treadmill decelerations, as this perturbation paradigm has been shown to affect one’s ability to maintain their center of mass (CoM) within their base of support (BoS) [24,25]. MoS_AP_ considers the body’s extrapolated CoM (xCoM), which is calculated using the CoM velocity (*v*_CoM_) and the relative speed of the treadmill (*v*_treadmill_) and $\omega_{\text{0}}$ in Eq. 1, where $\omega_{\text{0}}=\;\sqrt{g/l}$, where g = 9.81 m/s^2^ and *l* is the leg length. MoS_ML_ does not correct xCoM with the speed of the treadmi*ll* (Eq. 2). We defined leg length as the average distance between the sacral (S2) marker and the heel marker at each heel-strike.


$$\begin{array}{c} {xCoM}_{AP}={CoM}_{AP}+({vCoM}_{AP}+v_{treadmill})/\omega_{\text{0}} \end{array}$$ (1)

$$\begin{array}{c} {xCoM}_{ML}={CoM}_{ML}+{vCoM}_{ML}/\omega_{\text{0}} \end{array}$$ (2)

The anterior BoS was defined as the position of the first metatarsal marker and the mediolateral BoS was defined as the position of the fifth metatarsal marker. The CoM was calculated as the average position of the four anterior-posterior superior iliac spine markers, which has been used extensively in previous works [26–28]. MoS_AP_ and MoS_ML_ were calculated, as shown in Eq. 3, at the instant of the heel-strike following the deceleration as the distance between xCoM and the anterior and lateral BoS, respectively. A smaller MoS_ML_ indicates that the xCoM is closer to the lateral boundary of the stepping foot base of support, thus implying more instability. Conversely, a negative MoS_AP_ in the direction of travel has been deemed to imply greater instantaneous instability [29].


$$\begin{array}{c} MoS=BoS-xCoM \end{array}$$ (3)

To quantify lateral reactive stepping performance, we chose to analyze participants’ reaction time and error in foot placement. Using previously published methods [30], we calculated the magnitude of foot placement error as the lateral distance between the heel marker and the target line at the instant of ipsilateral heel strike after a lateral target appeared. The normal swing trajectory was calculated as the average swing trajectory of the heel during unperturbed walking trials. To remove any lateral offset and ensure toe-off occurred at the same lateral position, we subtracted the difference in the heel marker’s lateral position at toe-off prior to stepping toward the target and the average lateral position at toe-off during the unperturbed walking trial. The reaction time was calculated as the time at which the lateral position diverged from the normal trajectory by 2 standard deviations after toe-off [30]. To calculate error in foot placement (in meters), we calculated the lateral distance between the heel marker and the target at the instant of targeted heel strike.

### 2.3. Statistical analysis

To validate our protocol was successful in artificially inducing lordosis and kyphosis, a one-way repeated measures ANOVA compared head and neck angles with bracing condition as a within-subject factor in SPSS Statistics (IBM, Armonk, NY, USA) [31]. Tukey’s post-hoc pairwise comparisons were completed for those outcomes with significant main effects. For MoS_AP_, we conducted a two-way repeated measures ANOVA with bracing condition and perturbation (i.e., unperturbed vs. perturbed) as within-subject factors. For lateral reactive stepping performance (i.e., reaction time and foot placement error), we conducted a two-way repeated measures ANOVA with bracing condition and target distance (near/distant) as within-subject factors. Assumptions of normality were met using Shapiro-Wilk tests. As this was an exploratory study, we performed Tukey’s post-hoc pairwise comparisons regardless of significant main effects. An alpha level of 0.05 defined significance for this study. In addition, p-values ≤ 0.07 defined a statistical trend worthy of future study.

## Results

### 3.1. Protocol validation in artificially inducing kyphosis and lordosis

We found that our protocol successfully induced altered spinal postures using an adjustable universal cervical collar (Fig 2). During unperturbed walking, the head flexion ROM was significantly greater during unbraced walking than for any braced conditions (*p*-values≤0.012). During unperturbed walking, the lordotic condition resulted in significantly greater mean head (*p*-values≤0.023) and neck (*p*-values≤0.017) extension angles than all other conditions. Pairwise comparisons also revealed significantly different mean neck angles between unbraced and kyphosis and kyphosis and neutral bracing, with artificially induced kyphosis restricting the natural neck flexion (*p-*values≤0.014). Average step widths during the unperturbed trial are shown in Table 1 for quantitative comparisons of the effects of bracing conditions on step width (m). We found no statistical differences across conditions (main effect, p = 0.054).

**Table 1:** Average unperturbed step width across all bracing conditions.

Bracing Condition	Unbraced	Kyphosis	Neutral	Lordosis
**Step Width (m)**	0.17 ± 0.03	0.16 ± 0.03	0.17 ± 0.03	0.17 ± 0.04

The average ± standard deviation unperturbed step width (m) for all bracing conditions.

### 3.2. Margin of stability in response to treadmill belt decelerations

**Fig 3:**
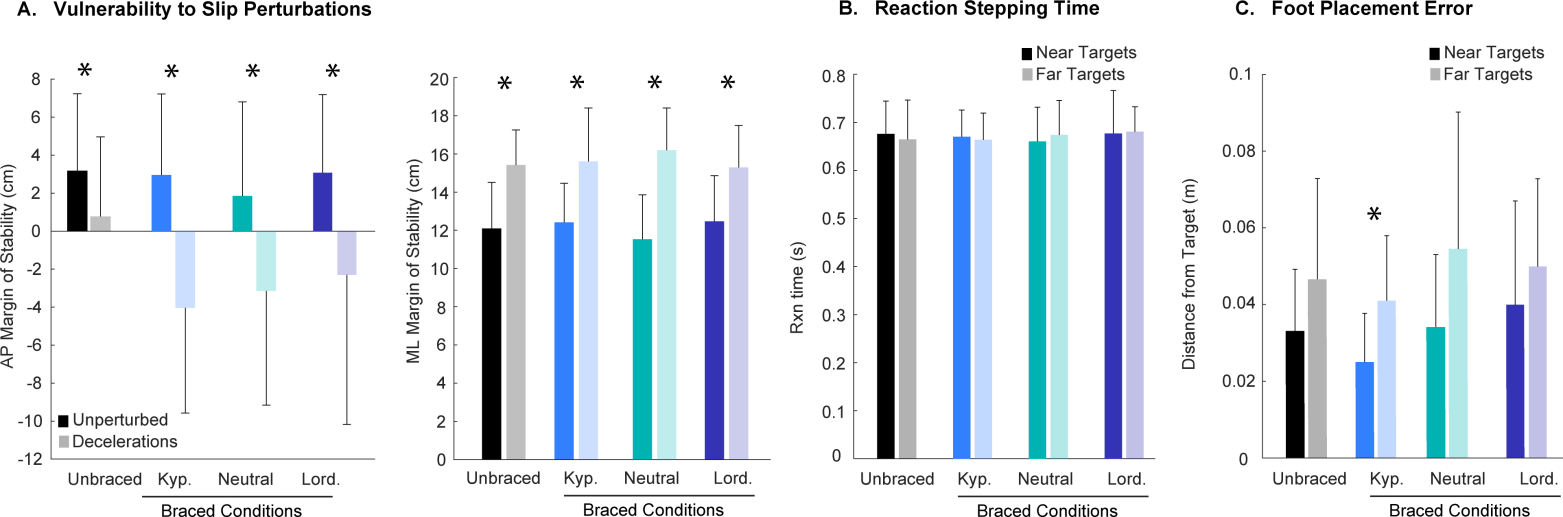
Anteroposterior (MoS_AP_) and mediolateral (MoS_ML_) margins of stability (cm), reactive stepping time (s), and foot placement error (m). Average values for normal, unperturbed walking are shown for each bracing condition in dark colors. Average values at the instant of heel strike following treadmill decelerations for each bracing condition are shown in light colors. (B) Reactive stepping time (seconds) for near (dark colors) and distant (light colors) targets for all four bracing conditions. (C) Foot placement error (m) for near (dark colors) and distant (light colors) targets for all four bracing conditions. Asterisks (*) denote significant differences within bracing conditions.

Spinal condition had no significant main effect on MoS_AP_ or MoS_ML_. However, our exploratory pairwise comparisons revealed a significant difference in MoS_AP_ between unbraced and neutral bracing independent of perturbation condition (p = 0.020). We found that perturbations had a significant main effect on MoS_AP_ (*p* = 0.001) and MoS_ML_ (*p* < 0.001) (Fig 3A-B). Compared to unperturbed walking, treadmill belt decelerations resulted in significantly smaller MoS_AP_ (p ≤ 0.010) but significantly larger MoS_ML_ (p ≤ 0.002) for all conditions. Qualitatively, we observed negative MoS_AP_ values in response to treadmill decelerations only for braced conditions; MoS_AP_ decreased but remained positive during the unbraced condition.

### 3.3. Reaction time and foot placement error

We found no significant main effects of target distance, bracing condition, or an interaction effect in reaction time during the reactive stepping task (*p* = 0.811, *p* = 0.905, *p* = 0.819, respectively) (Fig 3C). We found a significant main effect of target distance (*p* = 0.005), but not for bracing (*p* = 0.276) or condition × target distance interaction (p = 0.395) for foot placement accuracy. Pairwise comparisons revealed that foot placement accuracy was significantly different between near and distant targets for the kyphotic bracing condition (*p* = 0.002).

## Discussion

This study was designed to test the hypotheses that inducing altered cervical postures (i.e., kyphosis and lordosis) would elicit larger susceptibility to treadmill-induced slip perturbations and reduce lateral reactive stepping performance compared to walking with full cervical mobility and neutral immobilization. Our experimental manipulations were successful in emulating altered cervical spine postures common to patient populations. We accept our hypothesis that altered cervical postures, such as lordosis and kyphosis, as well as cervical immobilization, increase susceptibility to walking balance challenges, evidenced herein by negative MoS_AP_ in response to rapid treadmill belt decelerations, which is indicative of instability. We also partially accept our hypothesis that cervical spine deformities reduce lateral reactive stepping performance. Although bracing did not increase foot placement errors compared to unbraced walking, only for kyphotic bracing did performance decreased with target distance. Ultimately, this study conducted in healthy young controls serves as a foundation for further studies in patient populations with cervical spine deformities and disorders such as cervical spondylotic myelopathy.

We used margin of stability and treadmill-induced slip perturbations in this study to quantify the effects of cervical immobilization and posture on walking balance. We hypothesized that MoS_AP_ would decrease (i.e., become more negative) in response to treadmill belt decelerations, with larger effects for braced than unbraced walking. Our statistical analyses did support this hypothesis. In the direction of movement, a negative value for MoS_AP_ implies instantaneous instability at the instant of foot contact during walking [29]. During unperturbed walking, MoS_AP_ remained positive on average. Indeed, only for braced conditions did perturbations precipitate a negative MoS_AP_ on average. This finding alludes to a unique effect of cervical bracing and altered spinal postures on walking-related instability that warrants further study. Participants had significantly greater MoS_ML_ when responding to treadmill belt decelerations, independent of bracing condition. We suspect that by increasing mediolateral base of support during the recovery, participants reduced their susceptibility in the anterior-posterior direction.

There are potential clinical implications for follow-on studies designed to better understand the disproportionate prevalence of instability in patients with cervical immobilization or cervical spine deformities, particularly in patients. Our results suggest that altered cervical spine postures common to patients with diagnosed deformities may increase vulnerability to walking balance challenges relevant to their increased risk of falling. One implication would be that an indirect clinical consequence of treating altered cervical spine postures may be to mitigate the compounded risks walking-related instability. A second major implication of this finding is that prescription of cervical immobilization – a modality utilized for the treatment of a variety of cervical spine disorders – should be made in combination with educational materials and monitoring techniques tailored to patients and their caregivers to mitigate falls risks. Our findings should also motivate future work designed to investigate balance responses to other contexts of balance perturbations, as we have shown that such responses are task specific [25].

We partially accept our hypothesis that altered cervical postures would elicit reduced lateral reactive stepping performance compared to walking with full cervical mobility and neutral immobilization. Independent of target distance, bracing did not alter participants’ reaction time nor foot placement error during lateral reactive stepping. While this study investigated the biomechanical contribution of reduced cervical mobility on balance, we were not able to replicate additional deficits caused by DCM. We did not find differences in reaction times due to cervical immobilization in healthy participants. As previous studies found delayed reaction times in individuals with DCM [14,15], this suggests that neurological deficits may contribute more to reduced reaction times rather than cervical mobility alone. One explanation is that our younger adult control population did not have symptoms common to many spinal disorders, such neural or motor deficits [32]. Conversely, cervical spine posture did modify the effect of target distance on foot placement accuracy. Specifically, only when walking with cervical kyphosis did foot placement error increase for distant versus near targets. Anecdotally, participants were asked after completing the lateral reactive stepping task whether their strategy changed for any condition. Ten participants responded that they changed their trunk posture in the lordotic condition to compensate for neck extension. We suspect that this compensation may explain why we found no significant differences in foot placement error for the lordotic condition. Conversely, given the task requirements of seeing a target the belt surface, participants were unlikely to be compelled to change their trunk posture as a compensation when wearing the kyphotic brace. Thus, we suspect that a reduced capacity for transverse plane head and neck rotation may have reduced participants’ ability to see distant lateral targets.

There were several limitations in this study. One limitation is that this study induced altered cervical spine postures in healthy young individuals designed to emulate those common in patients with cervical deformities. Therefore, this study only focuses on the postural changes associated with cervical deformities and does not capture the effects of the associated neurological changes. However, our goal was to isolate the effects of postural changes on gait and stability without the presence of clinical comorbidities in individuals such as those with DCM. Thus, we contend that this study represents an important first step to establish a scientific foundation for future study. The prescribed altered spine postures were validated using head and neck angles, which may not fully reflect postural changes associated with clinical pathology. Inducing altered spinal postures in healthy young adults required using a cervical collar to prevent participants from changing their sagittal plane neck posture. Consequently, participants likely had decreased range of motion in the frontal and transverse planes that may not present in a clinical population. While this is consistent with neck bracing, this may have lessened the ecological validity of inducing kyphosis and lordosis. Additionally, participants anticipated some threat to their balance, particularly during treadmill deceleration trials. This limitation was mitigated by randomizing the leg on which the perturbation occurred. As we did not control for step width during our study, participants may have made adaptive changes in their baseline mediolateral margins of stability with potential effects on the ecological validity of our between-condition comparisons. Therefore, it is important to interpret our results in the context of these known limitations. Lastly, while using the pelvis markers to estimate the location of the body’s CoM is a well-established surrogate [26–28], this likely introduced small errors in this calculation.

In conclusion, we found that altered cervical spine postures, such as lordosis and kyphosis, and cervical immobilization increase susceptibility to walking balance challenges. Our results suggest that altered cervical spine postures common to patients with diagnosed deformities may increase their susceptibility to balance challenges relevant to precipitating a fall in the community.
