# Supplementary material for: The effects of cervical spine posture on susceptibility to walking balance perturbations
Source: PLoS One. 2026 Aug 5;21(8):e0354125. doi: 10.1371/journal.pone.0354125 (PMC13440797; doi:10.1371/journal.pone.0354125)
Supplement: Supplementary file 1 [file pone.0354125.s002.docx]

**1. Please ensure that your manuscript meets PLOS ONE's style requirements, including those for file naming.**

We have updated our manuscript to meet the PLOS ONE style requirements, including the title page, manuscript body, and file names.

**2. We note that the grant information you provided in the ‘Funding Information’ and ‘Financial Disclosure’ sections do not match. When you resubmit, please ensure that you provide the correct grant numbers for the awards you received for your study in the ‘Funding Information’ section.**

We have corrected our financial disclosure and funding information responses to match.

**3. In the online submission form you indicate that your data is not available for proprietary reasons and have provided a contact point for accessing this data. Please note that your current contact point is a co-author on this manuscript. According to our Data Policy, the contact point must not be an author on the manuscript and must be an institutional contact, ideally not an individual. Please revise your data statement to a non-author institutional point of contact, such as a data access or ethics committee, and send this to us via return email. Please also include contact information for the third party organization, and please include the full citation of where the data can be found.**

We have now published our data on Figshare here: [https://doi.org/10.15139/S3/V0ERXJ](https://doi.org/10.6084/m9.figshare.32476677)

**4. Your abstract cannot contain citations. Please only include citations in the body text of the manuscript, and ensure that they remain in ascending numerical order on first mention.**

We have removed citations from the abstract and all citations in the body of the manuscript are in ascending numerical order.

**5. Please include your full ethics statement in the ‘Methods’ section of your manuscript file. In your statement, please include the full name of the IRB or ethics committee who approved or waived your study, as well as whether or not you obtained informed written or verbal consent. If consent was waived for your study, please include this information in your statement as well.**

Our ethics statement has been updated to include the full name of the IRB committee and that we obtained written consent: “Prior to data collection, participants provided written informed consent and the study was approved by the University of North Carolina Biomedical Sciences Institutional Review Board (Study #21-3091). Recruitment began April 15, 2022 and concluded June 18, 2022” (Lines 91-94).

**6. If the reviewer comments include a recommendation to cite specific previously published works, please review and evaluate these publications to determine whether they are relevant and should be cited. There is no requirement to cite these works unless the editor has indicated otherwise.**

We have evaluated the suggested papers to include and found them relevant to our work. They are now included in our references.

**7. Please review your reference list to ensure that it is complete and correct. If you have cited papers that have been retracted, please include the rationale for doing so in the manuscript text, or remove these references and replace them with relevant current references. Any changes to the reference list should be mentioned in the rebuttal letter that accompanies your revised manuscript. If you need to cite a retracted article, indicate the article’s retracted status in the References list and also include a citation and full reference for the retraction notice.**

We have reviewed and our reference list is accurate as reported and consistent with these policies.

**Reviewer 1 comments**

**Cervical spondylotic myelopathy is a surgical pathology unless spontaneous movement of the myelomalacia region is lost and the patient's medical problems are at an acceptable level. A short-term collar may be needed after surgery. In my opinion, the authors have touched upon a very important issue. We use a neck brace that immobilizes the neck for every patient with neck pain. This study shows us that, not only in CSM patients but also in degenerative spine patients, pre-treatment gait analysis should be performed to determine whether or not to prescribe a cervical collar, or to warn the patient accordingly. Furthermore, new collar designs can be developed in the future.**

We thank the reviewer for their support and acknowledgement of the value added by our work.

**Reviewer 2 comments.
The authors present an interesting study examining the effects of neck postural changes (due to variations in immobilization and bracing) on balance responses in cervical spondylotic myelopathy. The experimentation, results, and conclusions appear technically sound. As such, I think this paper presents a substantial contribution to the literature on balance impairments in cervical myelopathy. I do have some concerns about presentation that should be addressed.**

**I have no major concerns.**

We thank the reviewer for their insightful comments and suggestions to improve our manuscript. We have implemented changes that we feel have strengthened our manuscript.

**Minor concerns:

One issue that impacts the quality of the paper and it's potential impact is the usage of older terminology (e.g. cervical spondylotic myelopathy, [CSM]) rather than more recently accepted terminology (degenerative cervical myelopathy, [DCM]). The authors are encouraged to read Davies et al. 2024 (PMID: 35769029) for consideration of terminology. While the usage of CSM is not inherently wrong as it is the legacy term, it's use along with a reference list containing only studies using CSM suggest the authors may not be aware of the parallel terminology. This appears to have resulted in the authors missing some key papers on perturbation-based balance training and biomechanical responses to perturbations in DCM. Some of the perturbation literature in DCM that appears to have been missed includes Cheng et al. 2020 (PMID: 32154235 ), Boerger et al 2022 (PMID: 35080466) and Boerger et al. 2024 (PMID: 38412683). This issue appears to have resulted in the incorrectly proposing (for example at line 68 and similar language) that perturbation-based balance assessments have not been performed in this population. Notably, the present paper gives important context to the previously mentioned papers. It is interesting that simply immobilizing the head results in changes in balance responses.**

We thank the reviewer for their insights on accepted terminology. Upon their suggestion, we have revised our manuscript to use degenerative cervical myelopathy (DCM) instead of cervical spondylotic myelopathy (CSM) in all cases throughout our manuscript.

We also thank the reviewer for providing relevant publications on deploying perturbations in individuals with DLB. We have updated our introduction on Lines 67-69 to reflect these and now read: “While perturbation-based balance training has been applied [1], and perturbation-based assessments have characterized balance impairments in individuals DCM [2, 3], the effects of cervical posture on balance has yet to be objectively quantified, even in otherwise healthy individuals.” **Consistent with my above comment, I would recommend the authors include "degenerative cervical myelopathy" as a keyword if they chose to not use it in the title or abstract. This will help with indexing the term and increase the impact of the present paper as Medline filters do not consider the terms interchangeable at this time.**

We have updated our list of keywords as recommended to include “degenerative cervical myelopathy.”

**While it is acknowledged in the limitations, there are important distinctions between the post perturbation balance responses of people with DCM that are not explained by immobilization and these should be more accurately described in the discussion. Young adults with immobilization did not have changes in response times, however, that was a key observation of Boerger et al 2022 and Boerger et al 2024. See also Nardone et al. 2008 (PMID: 18758671). Therefore, the present study represents a limited model of the functional impact of DCM on balance. It is, however, a very useful study for the broader theoretical framework of what contributes to balance impairments in DCM and that some of the impairments may be related to underlying spondylotic degeneration alone.**

We thank the reviewer for this important point and have revised the discussion to more explicitly acknowledge that while immobilization captures the biomechanical constraints of DCM, it does not capture other constraints caused by DCM, such as impaired reaction times. We have revised our discussion to include the following: “While this study investigated the biomechanical contribution of reduced cervical mobility on balance, we were not able to replicate additional deficits caused by DCM. We did not find differences in reaction times due to cervical immobilization in healthy participants. As previous studies found delayed reaction times in individuals with DCM [2, 3], this suggests that neurological deficits may contribute more to reduced reaction times rather than cervical mobility alone” (Lines 277-281).

**As DCM induces increases in step width, it is unclear why the authors chose to not explicitly report alterations in baseline step width and MOS in the collar conditions as those would add to the validity of the model as a model of DCM.**

We have now included the average step with during the unperturbed walking trials for all four bracing conditions. We have included this in our methods section on Lines 152-153: “We also calculated the average step width during unperturbed trials for all four bracing conditions.” The results are included as follows:

“Average step widths during the unperturbed trial are shown in Table 1 for quantitative comparisons of the effects of bracing conditions on step width (m). We found no statistical differences across conditions (main effect, p=0.054).

**Table 1. Average unperturbed step width across all bracing conditions.**

The average ± standard deviation unperturbed step width (m) for all bracing conditions.” (Lines 208-214).

| **Bracing Condition** | **Unbraced** | **Kyphosis** | **Neutral** | **Lordosis** |
| --- | --- | --- | --- | --- |
| **Step Width (m)** | 0.17±0.03 | 0.16±0.03 | 0.17±0.03 | 0.17±0.04 |

**The authors appear to not be in compliance with data sharing policies of the journal. This should be remedied.**

We have now published our data on Figshare here: [https://doi.org/10.15139/S3/V0ERXJ](https://doi.org/10.6084/m9.figshare.32476677)

**References**

1.	Cheng YS, Chien, A., Lai, D. M., Lee, Y. Y., Cheng, C. H., Wang, S. F., Chang, Y. J., Wang, J. L., & Hsu, W. L. . Perturbation-Based Balance Training in Postoperative Individuals With Degenerative Cervical Myelopathy. Frontiers in bioengineering and biotechnology. 2020;8(108).

2.	Boerger TF, McGinn, L., Wang, M. C., Schmit, B. D., & Hyngstrom, A. S. Degenerative cervical myelopathy delays responses to lateral balance perturbations regardless of predictability. Journal of neurophysiology. 2022;127(3).

3.	Boerger TF, McGinn, L., Bellman, M., Wang, M. C., Schmit, B. D., & Hyngstrom, A. S. People with degenerative cervical myelopathy have impaired reactive balance during walking. Gait & posture. 2024;109.
