# Supplementary material for: The effects of cervical spine posture on susceptibility to walking balance perturbations
Source: PLoS One. 2026 Aug 5;21(8):e0354125. doi: 10.1371/journal.pone.0354125 (PMC13440797; doi:10.1371/journal.pone.0354125)
Supplement: Peer review history

# Peer review: Decision Letter 0

**DOI:** 10.1371/journal.pone.0354125.r001

**Authors:** Isamu Miura

17 Mar 2026

PONE-D-25-18010

The effects of cervical spine posture on susceptibility to walking balance perturbations

PLOS One

Dear Dr. Franz,

Thank you for submitting your manuscript to PLOS ONE. After careful consideration, we feel that it has merit but does not fully meet PLOS ONE’s publication criteria as it currently stands. Therefore, we invite you to submit a revised version of the manuscript that addresses the points raised during the review process.

Please submit your revised manuscript by May 01 2026 11:59PM. If you will need more time than this to complete your revisions, please reply to this message or contact the journal office at plosone@plos.org. When you're ready to submit your revision, log on to https://www.editorialmanager.com/pone/ and select the 'Submissions Needing Revision' folder to locate your manuscript file.

Please include the following items when submitting your revised manuscript:

- A letter that responds to each point raised by the academic editor and reviewer(s). You should upload this letter as a separate file labeled 'Response to Reviewers'.
- A marked-up copy of your manuscript that highlights changes made to the original version. You should upload this as a separate file labeled 'Revised Manuscript with Track Changes'.
- An unmarked version of your revised paper without tracked changes. You should upload this as a separate file labeled 'Manuscript'.

-->If you would like to make changes to your financial disclosure, please include your updated statement in your cover letter. Guidelines for resubmitting your figure files are available below the reviewer comments at the end of this letter.

If applicable, we recommend that you deposit your laboratory protocols in protocols.io to enhance the reproducibility of your results. Protocols.io assigns your protocol its own identifier (DOI) so that it can be cited independently in the future. For instructions see: https://journals.plos.org/plosone/s/submission-guidelines#loc-laboratory-protocols. Additionally, PLOS ONE offers an option for publishing peer-reviewed Lab Protocol articles, which describe protocols hosted on protocols.io. Read more information on sharing protocols at https://plos.org/protocols?utm_medium=editorial-email&utm_source=authorletters&utm_campaign=protocols.

We look forward to receiving your revised manuscript.

Kind regards,

Isamu Miura

Academic Editor

PLOS One

**Journal Requirements:**

When submitting your revision, we need you to address these additional requirements.

1. Please ensure that your manuscript meets PLOS ONE's style requirements, including those for file naming. The PLOS ONE style templates can be found at

https://journals.plos.org/plosone/s/file?id=wjVg/PLOSOne_formatting_sample_main_body.pdf and

https://journals.plos.org/plosone/s/file?id=ba62/PLOSOne_formatting_sample_title_authors_affiliations.pdf

When submitting your revision, we need you to address these additional requirements.

1. Please ensure that your manuscript meets PLOS ONE's style requirements, including those for file naming. The PLOS ONE style templates can be found at

https://journals.plos.org/plosone/s/file?id=wjVg/PLOSOne_formatting_sample_main_body.pdf and

https://journals.plos.org/plosone/s/file?id=ba62/PLOSOne_formatting_sample_title_authors_affiliations.pdf

2. We note that the grant information you provided in the ‘Funding Information’ and ‘Financial Disclosure’ sections do not match.

When you resubmit, please ensure that you provide the correct grant numbers for the awards you received for your study in the ‘Funding Information’ section.

3. In the online submission form you indicate that your data is not available for proprietary reasons and have provided a contact point for accessing this data. Please note that your current contact point is a co-author on this manuscript. According to our Data Policy, the contact point must not be an author on the manuscript and must be an institutional contact, ideally not an individual. Please revise your data statement to a non-author institutional point of contact, such as a data access or ethics committee, and send this to us via return email. Please also include contact information for the third party organization, and please include the full citation of where the data can be found.

4. Your abstract cannot contain citations. Please only include citations in the body text of the manuscript, and ensure that they remain in ascending numerical order on first mention.

5. Please include your full ethics statement in the ‘Methods’ section of your manuscript file. In your statement, please include the full name of the IRB or ethics committee who approved or waived your study, as well as whether or not you obtained informed written or verbal consent. If consent was waived for your study, please include this information in your statement as well.

6. If the reviewer comments include a recommendation to cite specific previously published works, please review and evaluate these publications to determine whether they are relevant and should be cited. There is no requirement to cite these works unless the editor has indicated otherwise.

7. Please review your reference list to ensure that it is complete and correct. If you have cited papers that have been retracted, please include the rationale for doing so in the manuscript text, or remove these references and replace them with relevant current references. Any changes to the reference list should be mentioned in the rebuttal letter that accompanies your revised manuscript. If you need to cite a retracted article, indicate the article’s retracted status in the References list and also include a citation and full reference for the retraction notice.

**Additional Editor Comments:**

Reviewer 1 comments.

Cervical spondylotic myelopathy is a surgical pathology unless spontaneous movement of the myelomalacia region is lost and the patient's medical problems are at an acceptable level. A short-term collar may be needed after surgery. In my opinion, the authors have touched upon a very important issue. We use a neck brace that immobilizes the neck for every patient with neck pain. This study shows us that, not only in CSM patients but also in degenerative spine patients, pre-treatment gait analysis should be performed to determine whether or not to prescribe a cervical collar, or to warn the patient accordingly. Furthermore, new collar designs can be developed in the future.

Reviewer 2 comments.

The authors present an interesting study examining the effects of neck postural changes (due to variations in immobilization and bracing) on balance responses in cervical spondylotic myelopathy. The experimentation, results, and conclusions appear technically sound. As such, I think this paper presents a substantial contribution to the literature on balance impairments in cervical myelopathy. I do have some concerns about presentation that should be addressed.

I have no major concerns.

Minor concerns:

One issue that impacts the quality of the paper and it's potential impact is the usage of older terminology (e.g. cervical spondylotic myelopathy, [CSM]) rather than more recently accepted terminology (degenerative cervical myelopathy, [DCM]). The authors are encouraged to read Davies et al. 2024 (PMID: 35769029) for consideration of terminology. While the usage of CSM is not inherently wrong as it is the legacy term, it's use along with a reference list containing only studies using CSM suggest the authors may not be aware of the parallel terminology. This appears to have resulted in the authors missing some key papers on perturbation-based balance training and biomechanical responses to perturbations in DCM. Some of the perturbation literature in DCM that appears to have been missed includes Cheng et al. 2020 (PMID: 32154235 ), Boerger et al 2022 (PMID: 35080466) and Boerger et al. 2024 (PMID: 38412683). This issue appears to have resulted in the incorrectly proposing (for example at line 68 and similar language) that perturbation-based balance assessments have not been performed in this population. Notably, the present paper gives important context to the previously mentioned papers. It is interesting that simply immobilizing the head results in changes in balance responses.

Consistent with my above comment, I would recommend the authors include "degenerative cervical myelopathy" as a keyword if they chose to not use it in the title or abstract. This will help with indexing the term and increase the impact of the present paper as Medline filters do not consider the terms interchangeable at this time.

While it is acknowledged in the limitations, there are important distinctions between the post perturbation balance responses of people with DCM that are not explained by immobilization and these should be more accurately described in the discussion. Young adults with immobilization did not have changes in response times, however, that was a key observation of Boerger et al 2022 and Boerger et al 2024. See also Nardone et al. 2008 (PMID: 18758671). Therefore, the present study represents a limited model of the functional impact of DCM on balance. It is, however, a very useful study for the broader theoretical framework of what contributes to balance impairments in DCM and that some of the impairments may be related to underlying spondylotic degeneration alone.

As DCM induces increases in step width, it is unclear why the authors chose to not explicitly report alterations in baseline step width and MOS in the collar conditions as those would add to the validity of the model as a model of DCM.

The authors appear to not be in compliance with data sharing policies of the journal. This should be remedied.

[Note: HTML markup is below. Please do not edit.]

Reviewers' comments:

Reviewer's Responses to Questions

**Comments to the Author**

1. Is the manuscript technically sound, and do the data support the conclusions?

The manuscript must describe a technically sound piece of scientific research with data that supports the conclusions. Experiments must have been conducted rigorously, with appropriate controls, replication, and sample sizes. The conclusions must be drawn appropriately based on the data presented.

Reviewer #1: Yes

Reviewer #2: Yes

**********

2. Has the statistical analysis been performed appropriately and rigorously?

Reviewer #1: N/A

Reviewer #2: Yes

**********

3. Have the authors made all data underlying the findings in their manuscript fully available?

The PLOS Data policy requires authors to make all data underlying the findings described in their manuscript fully available without restriction, with rare exception (please refer to the Data Availability Statement in the manuscript PDF file). The data should be provided as part of the manuscript or its supporting information, or deposited to a public repository. For example, in addition to summary statistics, the data points behind means, medians and variance measures should be available. If there are restrictions on publicly sharing data—e.g. participant privacy or use of data from a third party—those must be specified.

Reviewer #1: Yes

Reviewer #2: No

**********

4. Is the manuscript presented in an intelligible fashion and written in standard English?

PLOS ONE does not copyedit accepted manuscripts, so the language in submitted articles must be clear, correct, and unambiguous. Any typographical or grammatical errors should be corrected at revision, so please note any specific errors here.

Reviewer #1: Yes

Reviewer #2: Yes

**********

5. Review Comments to the Author

Please use the space provided to explain your answers to the questions above. You may also include additional comments for the author, including concerns about dual publication, research ethics, or publication ethics. (Please upload your review as an attachment if it exceeds 20,000 characters)

Reviewer #1: Cervical spondylotic myelopathy is a surgical pathology unless spontaneous movement of the myelomalacia region is lost and the patient's medical problems are at an acceptable level. A short-term collar may be needed after surgery. In my opinion, the authors have touched upon a very important issue. We use a neck brace that immobilizes the neck for every patient with neck pain. This study shows us that, not only in CSM patients but also in degenerative spine patients, pre-treatment gait analysis should be performed to determine whether or not to prescribe a cervical collar, or to warn the patient accordingly. Furthermore, new collar designs can be developed in the future.

Reviewer #2: The authors present an interesting study examining the effects of neck postural changes (due to variations in immobilization and bracing) on balance responses in cervical spondylotic myelopathy. The experimentation, results, and conclusions appear technically sound. As such, I think this paper presents a substantial contribution to the literature on balance impairments in cervical myelopathy. I do have some concerns about presentation that should be addressed.

I have no major concerns.

Minor concerns:

One issue that impacts the quality of the paper and it's potential impact is the usage of older terminology (e.g. cervical spondylotic myelopathy, [CSM]) rather than more recently accepted terminology (degenerative cervical myelopathy, [DCM]). The authors are encouraged to read Davies et al. 2024 (PMID: 35769029) for consideration of terminology. While the usage of CSM is not inherently wrong as it is the legacy term, it's use along with a reference list containing only studies using CSM suggest the authors may not be aware of the parallel terminology. This appears to have resulted in the authors missing some key papers on perturbation-based balance training and biomechanical responses to perturbations in DCM. Some of the perturbation literature in DCM that appears to have been missed includes Cheng et al. 2020 (PMID: 32154235 ), Boerger et al 2022 (PMID: 35080466) and Boerger et al. 2024 (PMID: 38412683). This issue appears to have resulted in the incorrectly proposing (for example at line 68 and similar language) that perturbation-based balance assessments have not been performed in this population. Notably, the present paper gives important context to the previously mentioned papers. It is interesting that simply immobilizing the head results in changes in balance responses.

Consistent with my above comment, I would recommend the authors include "degenerative cervical myelopathy" as a keyword if they chose to not use it in the title or abstract. This will help with indexing the term and increase the impact of the present paper as Medline filters do not consider the terms interchangeable at this time.

While it is acknowledged in the limitations, there are important distinctions between the post perturbation balance responses of people with DCM that are not explained by immobilization and these should be more accurately described in the discussion. Young adults with immobilization did not have changes in response times, however, that was a key observation of Boerger et al 2022 and Boerger et al 2024. See also Nardone et al. 2008 (PMID: 18758671). Therefore, the present study represents a limited model of the functional impact of DCM on balance. It is, however, a very useful study for the broader theoretical framework of what contributes to balance impairments in DCM and that some of the impairments may be related to underlying spondylotic degeneration alone.

As DCM induces increases in step width, it is unclear why the authors chose to not explicitly report alterations in baseline step width and MOS in the collar conditions as those would add to the validity of the model as a model of DCM.

The authors appear to not be in compliance with data sharing policies of the journal. This should be remedied.

**********

6. PLOS authors have the option to publish the peer review history of their article (what does this mean?). If published, this will include your full peer review and any attached files.

If you choose “no”, your identity will remain anonymous but your review may still be made public.

**Do you want your identity to be public for this peer review?**  For information about this choice, including consent withdrawal, please see our Privacy Policy.

Reviewer #1: **Yes:**  Ali Fahir Ozer

Reviewer #2: No

**********

[NOTE: If reviewer comments were submitted as an attachment file, they will be attached to this email and accessible via the submission site. Please log into your account, locate the manuscript record, and check for the action link "View Attachments". If this link does not appear, there are no attachment files.]

To ensure your figures meet our technical requirements, please review our figure guidelines: https://journals.plos.org/plosone/s/figures

You may also use PLOS’s free figure tool, NAAS, to help you prepare publication quality figures: https://journals.plos.org/plosone/s/figures#loc-tools-for-figure-preparation.

NAAS will assess whether your figures meet our technical requirements by comparing each figure against our figure specifications.

# Author comment: Author response to Decision Letter 1

**DOI:** 10.1371/journal.pone.0354125.r002

3 Jun 2026

1. Please ensure that your manuscript meets PLOS ONE's style requirements, including those for file naming.

We have updated our manuscript to meet the PLOS ONE style requirements, including the title page, manuscript body, and file names.

2. We note that the grant information you provided in the ‘Funding Information’ and ‘Financial Disclosure’ sections do not match. When you resubmit, please ensure that you provide the correct grant numbers for the awards you received for your study in the ‘Funding Information’ section.

We have corrected our financial disclosure and funding information responses to match.

3. In the online submission form you indicate that your data is not available for proprietary reasons and have provided a contact point for accessing this data. Please note that your current contact point is a co-author on this manuscript. According to our Data Policy, the contact point must not be an author on the manuscript and must be an institutional contact, ideally not an individual. Please revise your data statement to a non-author institutional point of contact, such as a data access or ethics committee, and send this to us via return email. Please also include contact information for the third party organization, and please include the full citation of where the data can be found.

We have now published our data on figshare here: https://doi.org/10.15139/S3/V0ERXJ

4. Your abstract cannot contain citations. Please only include citations in the body text of the manuscript, and ensure that they remain in ascending numerical order on first mention.

We have removed citations from the abstract and all citations in the body of the manuscript are in ascending numerical order.

5. Please include your full ethics statement in the ‘Methods’ section of your manuscript file. In your statement, please include the full name of the IRB or ethics committee who approved or waived your study, as well as whether or not you obtained informed written or verbal consent. If consent was waived for your study, please include this information in your statement as well.

Our ethics statement has been updated to include the full name of the IRB committee and that we obtained written consent: “Prior to data collection, participants provided written informed consent and the study was approved by the University of North Carolina Biomedical Sciences Institutional Review Board (Study #21-3091). Recruitment began April 15, 2022 and concluded June 18, 2022” (Lines 91-94).

6. If the reviewer comments include a recommendation to cite specific previously published works, please review and evaluate these publications to determine whether they are relevant and should be cited. There is no requirement to cite these works unless the editor has indicated otherwise.

We have evaluated the suggested papers to include and found them relevant to our work. They are now included in our references.

7. Please review your reference list to ensure that it is complete and correct. If you have cited papers that have been retracted, please include the rationale for doing so in the manuscript text, or remove these references and replace them with relevant current references. Any changes to the reference list should be mentioned in the rebuttal letter that accompanies your revised manuscript. If you need to cite a retracted article, indicate the article’s retracted status in the References list and also include a citation and full reference for the retraction notice.

We have reviewed and our reference list is accurate as reported and consistent with these policies.

Reviewer 1 comments

Cervical spondylotic myelopathy is a surgical pathology unless spontaneous movement of the myelomalacia region is lost and the patient's medical problems are at an acceptable level. A short-term collar may be needed after surgery. In my opinion, the authors have touched upon a very important issue. We use a neck brace that immobilizes the neck for every patient with neck pain. This study shows us that, not only in CSM patients but also in degenerative spine patients, pre-treatment gait analysis should be performed to determine whether or not to prescribe a cervical collar, or to warn the patient accordingly. Furthermore, new collar designs can be developed in the future.

We thank the reviewer for their support and acknowledgement of the value added by our work.

Reviewer 2 comments.

The authors present an interesting study examining the effects of neck postural changes (due to variations in immobilization and bracing) on balance responses in cervical spondylotic myelopathy. The experimentation, results, and conclusions appear technically sound. As such, I think this paper presents a substantial contribution to the literature on balance impairments in cervical myelopathy. I do have some concerns about presentation that should be addressed.

I have no major concerns.

We thank the reviewer for their insightful comments and suggestions to improve our manuscript. We have implemented changes that we feel have strengthened our manuscript.

Minor concerns:

One issue that impacts the quality of the paper and it's potential impact is the usage of older terminology (e.g. cervical spondylotic myelopathy, [CSM]) rather than more recently accepted terminology (degenerative cervical myelopathy, [DCM]). The authors are encouraged to read Davies et al. 2024 (PMID: 35769029) for consideration of terminology. While the usage of CSM is not inherently wrong as it is the legacy term, it's use along with a reference list containing only studies using CSM suggest the authors may not be aware of the parallel terminology. This appears to have resulted in the authors missing some key papers on perturbation-based balance training and biomechanical responses to perturbations in DCM. Some of the perturbation literature in DCM that appears to have been missed includes Cheng et al. 2020 (PMID: 32154235 ), Boerger et al 2022 (PMID: 35080466) and Boerger et al. 2024 (PMID: 38412683). This issue appears to have resulted in the incorrectly proposing (for example at line 68 and similar language) that perturbation-based balance assessments have not been performed in this population. Notably, the present paper gives important context to the previously mentioned papers. It is interesting that simply immobilizing the head results in changes in balance responses.

We thank the reviewer for their insights on accepted terminology. Upon their suggestion, we have revised our manuscript to use degenerative cervical myelopathy (DCM) instead of cervical spondylotic myelopathy (CSM) in all cases throughout our manuscript.

We also thank the reviewer for providing relevant publications on deploying perturbations in individuals with DLB. We have updated our introduction on Lines 67-69 to reflect these and now read: “While perturbation-based balance training has been applied [1], and perturbation-based assessments have characterized balance impairments in individuals DCM [2, 3], the effects of cervical posture on balance has yet to be objectively quantified, even in otherwise healthy individuals.”

Consistent with my above comment, I would recommend the authors include "degenerative cervical myelopathy" as a keyword if they chose to not use it in the title or abstract. This will help with indexing the term and increase the impact of the present paper as Medline filters do not consider the terms interchangeable at this time.

We have updated our list of keywords as recommended to include “degenerative cervical myelopathy.”

While it is acknowledged in the limitations, there are important distinctions between the post perturbation balance responses of people with DCM that are not explained by immobilization and these should be more accurately described in the discussion. Young adults with immobilization did not have changes in response times, however, that was a key observation of Boerger et al 2022 and Boerger et al 2024. See also Nardone et al. 2008 (PMID: 18758671). Therefore, the present study represents a limited model of the functional impact of DCM on balance. It is, however, a very useful study for the broader theoretical framework of what contributes to balance impairments in DCM and that some of the impairments may be related to underlying spondylotic degeneration alone.

We thank the reviewer for this important point and have revised the discussion to more explicitly acknowledge that while immobilization captures the biomechanical constraints of DCM, it does not capture other constraints caused by DCM, such as impaired reaction times. We have revised our discussion to include the following: “While this study investigated the biomechanical contribution of reduced cervical mobility on balance, we were not able to replicate additional deficits caused by DCM. We did not find differences in reaction times due to cervical immobilization in healthy participants. As previous studies found delayed reaction times in individuals with DCM [2, 3], this suggests that neurological deficits may contribute more to reduced reaction times rather than cervical mobility alone” (Lines 277-281).

As DCM induces increases in step width, it is unclear why the authors chose to not explicitly report alterations in baseline step width and MOS in the collar conditions as those would add to the validity of the model as a model of DCM.

We have now included the average step with during the unperturbed walking trials for all four bracing conditions. We have included this in our methods section on Lines 152-153: “We also calculated the average step width during unperturbed trials for all four bracing conditions.” The results are included as follows:

“Average step widths during the unperturbed trial are shown in Table 1 for quantitative comparisons of the effects of bracing conditions on step width (m). We found no statistical differences across conditions (main effect, p=0.054).

Table 1. Average unperturbed step width across all bracing conditions.

The average ± standard deviation unperturbed step width (m) for all bracing conditions.” (Lines 208-214).

Bracing Condition Unbraced Kyphosis Neutral Lordosis

Step Width (m) 0.17±0.03 0.16±0.03 0.17±0.03 0.17±0.04

The authors appear to not be in compliance with data sharing policies of the journal. This should be remedied.

We have now published our data on figshare here: https://doi.org/10.15139/S3/V0ERXJ

References

1. Cheng YS, Chien, A., Lai, D. M., Lee, Y. Y., Cheng, C. H., Wang, S. F., Chang, Y. J., Wang, J. L., & Hsu, W. L. . Perturbation-Based Balance Training in Postoperative Individuals With Degenerative Cervical Myelopathy. Frontiers in bioengineering and biotechnology. 2020;8(108).

2. Boerger TF, McGinn, L., Wang, M. C., Schmit, B. D., & Hyngstrom, A. S. Degenerative cervical myelopathy delays responses to lateral balance perturbations regardless of predictability. Journal of neurophysiology. 2022;127(3).

3. Boerger TF, McGinn, L., Bellman, M., Wang, M. C., Schmit, B. D., & Hyngstrom, A. S. People with degenerative cervical myelopathy have impaired reactive balance during walking. Gait & posture. 2024;109.

Attachment Submitted filename: Response to Reviewers_v2.docx

# Peer review: Decision Letter 1

**DOI:** 10.1371/journal.pone.0354125.r003

**Authors:** Isamu Miura

5 Jul 2026

The effects of cervical spine posture on susceptibility to walking balance perturbations

PONE-D-25-18010R1

Dear Dr. Franz,

We’re pleased to inform you that your manuscript has been judged scientifically suitable for publication and will be formally accepted for publication once it meets all outstanding technical requirements.

Within one week, you’ll receive an e-mail detailing the required amendments. When these have been addressed, you’ll receive a formal acceptance letter and your manuscript will be scheduled for publication.

An invoice will be generated when your article is formally accepted. Please note, if your institution has a publishing partnership with PLOS and your article meets the relevant criteria, all or part of your publication costs will be covered. Please make sure your user information is up-to-date by logging into Editorial Manager at Editorial Manager® and clicking the ‘Update My Information' link at the top of the page. For questions related to billing, please contact billing support.

If your institution or institutions have a press office, please notify them about your upcoming paper to help maximize its impact. If they’ll be preparing press materials, please inform our press team as soon as possible -- no later than 48 hours after receiving the formal acceptance. Your manuscript will remain under strict press embargo until 2 pm Eastern Time on the date of publication. For more information, please contact onepress@plos.org.

Kind regards,

Isamu Miura

Academic Editor

PLOS One

Additional Editor Comments (optional):

All reviewer comments were addressed.

Reviewers' comments:

Reviewer's Responses to Questions

**Comments to the Author**

1. If the authors have adequately addressed your comments raised in a previous round of review and you feel that this manuscript is now acceptable for publication, you may indicate that here to bypass the “Comments to the Author” section, enter your conflict of interest statement in the “Confidential to Editor” section, and submit your "Accept" recommendation.

Reviewer #1: All comments have been addressed

Reviewer #2: All comments have been addressed

**********

2. Is the manuscript technically sound, and do the data support the conclusions?

The manuscript must describe a technically sound piece of scientific research with data that supports the conclusions. Experiments must have been conducted rigorously, with appropriate controls, replication, and sample sizes. The conclusions must be drawn appropriately based on the data presented.

Reviewer #1: Yes

Reviewer #2: Yes

**********

3. Has the statistical analysis been performed appropriately and rigorously?

Reviewer #1: N/A

Reviewer #2: Yes

**********

4. Have the authors made all data underlying the findings in their manuscript fully available?

The PLOS Data policy requires authors to make all data underlying the findings described in their manuscript fully available without restriction, with rare exception (please refer to the Data Availability Statement in the manuscript PDF file). The data should be provided as part of the manuscript or its supporting information, or deposited to a public repository. For example, in addition to summary statistics, the data points behind means, medians and variance measures should be available. If there are restrictions on publicly sharing data—e.g. participant privacy or use of data from a third party—those must be specified.

Reviewer #1: Yes

Reviewer #2: Yes

**********

5. Is the manuscript presented in an intelligible fashion and written in standard English?

PLOS ONE does not copyedit accepted manuscripts, so the language in submitted articles must be clear, correct, and unambiguous. Any typographical or grammatical errors should be corrected at revision, so please note any specific errors here.

Reviewer #1: Yes

Reviewer #2: Yes

**********

6. Review Comments to the Author

Please use the space provided to explain your answers to the questions above. You may also include additional comments for the author, including concerns about dual publication, research ethics, or publication ethics. (Please upload your review as an attachment if it exceeds 20,000 characters)

Reviewer #1: I believe the authors have touched upon a very important issue. This study shows us that pre-treatment gait analysis should be performed not only in patients with cervical spondylotic myelopathy but also in patients with degenerative spine diseases to decide whether to prescribe a neck brace or provide appropriate guidance. Furthermore, new neck brace designs could be developed in the future. This article is suitable for publication.

Reviewer #2: (No Response)

**********

7. PLOS authors have the option to publish the peer review history of their article (what does this mean?). If published, this will include your full peer review and any attached files.

If you choose “no”, your identity will remain anonymous but your review may still be made public.

**Do you want your identity to be public for this peer review?**  For information about this choice, including consent withdrawal, please see our Privacy Policy.

Reviewer #1: No

Reviewer #2: No

**********

# Editor report: Acceptance letter

**DOI:** 10.1371/journal.pone.0354125.r004

**Authors:** Isamu Miura

PONE-D-25-18010R1

PLOS One

Dear Dr. Franz,

I'm pleased to inform you that your manuscript has been deemed suitable for publication in PLOS One. Congratulations! Your manuscript is now being handed over to our production team.

At this stage, our production department will prepare your paper for publication. This includes ensuring the following:

* All references, tables, and figures are properly cited

* All relevant supporting information is included in the manuscript submission,

* There are no issues that prevent the paper from being properly typeset

You will receive further instructions from the production team, including instructions on how to review your proof when it is ready. Please keep in mind that we are working through a large volume of accepted articles, so please give us a few days to review your paper and let you know the next and final steps.

Lastly, if your institution or institutions have a press office, please let them know about your upcoming paper now to help maximize its impact. If they'll be preparing press materials, please inform our press team within the next 48 hours. Your manuscript will remain under strict press embargo until 2 pm Eastern Time on the date of publication. For more information, please contact onepress@plos.org.

You will receive an invoice from PLOS for your publication fee after your manuscript has reached the completed accept phase. If you receive an email requesting payment before acceptance or for any other service, this may be a phishing scheme. Learn how to identify phishing emails and protect your accounts at https://explore.plos.org/phishing.

If we can help with anything else, please email us at customercare@plos.org.

Thank you for submitting your work to PLOS ONE and supporting open access.

Kind regards,

PLOS ONE Editorial Office Staff

on behalf of

Dr. Isamu Miura

Academic Editor

PLOS One
